# Differentiation of glioma and solitary brain metastasis: a multi-parameter magnetic resonance imaging study using histogram analysis

**DOI:** 10.1186/s12885-024-12571-5

**Published:** 2024-07-05

**Authors:** Yifei Su, Rui Cheng, Jinxia Guo, Miaoqi Zhang, Junhao Wang, Hongming Ji, Chunhong Wang, Liangliang Hao, Yexin He, Cheng Xu

**Affiliations:** 1 The Neurosurgery Department of Shanxi Provincial People’s Hospital, Shanxi Medical University, Taiyuan, Shanxi 030012 PR China; 2 The Neurosurgery Department of Shanxi Provincial People’s Hospital, Taiyuan, Shanxi 030012 PR China; 3 GE Healthcare, Beijing, PR China; 4 Provincial Key Cultivation Laboratory of Intelligent Big Data Digital Neurosurgery of Shanxi Province, Taiyuan, Shanxi PR China; 5 The Radiology Department of Shanxi Provincial People’s Hospital, Taiyuan, Shanxi 030012 PR China

**Keywords:** Intravoxel incoherent motion, Amide proton transfer-weighted imaging, Glioma, Solitary brain metastasis, MRI

## Abstract

**Background:**

Differentiation of glioma and solitary brain metastasis (SBM), which requires biopsy or multi-disciplinary diagnosis, remains sophisticated clinically. Histogram analysis of MR diffusion or molecular imaging hasn’t been fully investigated for the differentiation and may have the potential to improve it.

**Methods:**

A total of 65 patients with newly diagnosed glioma or metastases were enrolled. All patients underwent DWI, IVIM, and APTW, as well as the T1W, T2W, T2FLAIR, and contrast-enhanced T1W imaging. The histogram features of apparent diffusion coefficient (ADC) from DWI, slow diffusion coefficient (Dslow), perfusion fraction (frac), fast diffusion coefficient (Dfast) from IVIM, and MTRasym@3.5ppm from APTWI were extracted from the tumor parenchyma and compared between glioma and SBM. Parameters with significant differences were analyzed with the logistics regression and receiver operator curves to explore the optimal model and compare the differentiation performance.

**Results:**

Higher ADC_kurtosis_ (*P* = 0.022), frac_kurtosis_ (*P*<0.001),and frac_skewness_ (*P*<0.001) were found for glioma, while higher (MTRasym@3.5ppm)_10_ (*P* = 0.045), frac_10_ (*P*<0.001),frac_90_ (*P* = 0.001), frac_mean_ (*P*<0.001), and frac_entropy_ (*P*<0.001) were observed for SBM. frac_kurtosis_ (OR = 0.431, 95%CI 0.256–0.723, *P* = 0.002) was independent factor for SBM differentiation. The model combining (MTRasym@3.5ppm)_10_, frac_10_, and frac_kurtosis_ showed an AUC of 0.857 (sensitivity: 0.857, specificity: 0.750), while the model combined with frac_10_ and frac_kurtosis_ had an AUC of 0.824 (sensitivity: 0.952, specificity: 0.591). There was no statistically significant difference between AUCs from the two models. (Z = -1.14, *P* = 0.25).

**Conclusions:**

The frac_10_ and frac_kurtosis_ in enhanced tumor region could be used to differentiate glioma and SBM and (MTRasym@3.5ppm)_10_ helps improving the differentiation specificity.

**Supplementary Information:**

The online version contains supplementary material available at 10.1186/s12885-024-12571-5.

## Background

Glioma and solitary brain metastasis (SBM) are neoplastic diseases with high morbidity and mortality worldwide [1, 2]. Different treatment strategies and clinical management are used for these two types of tumors. Maximum-safe-resection followed by chemoradiotherapy is recommended for glioma to reduce tumor size and acquire tumor tissue for identifying the grade [3]. For SBM, surgery is considered only when the number of lesions is less than 4 or there are high-risk or life-threatening clinical symptoms, such as intracranial hypertension, tumor apoplexy, increased edema, obstructive hydrocephalus, and so on. Moreover, brain radiotherapy is also recommended to improve the life quality of patients with SBM. [4]. Therefore, accurate differentiation of glioma and SBM is of crucial importance. Yet, clinically, patients with glioma and SBM may present with similar symptoms, including secondary epilepsy, dysfunction, and intracranial hypertension; thus, differentiation remains challenging.

Magnetic resonance imaging (MRI) is commonly used to diagnosis and differentiate brain neoplasms. Conventional MRIs such as T1, T2 weighted (T1W, T2W), and contrast-enhanced T1W (T1W + C) can identify well structural abnormalities such as mass size, shape and location, edema, ring-enhancing, and necrosis, as well as the degree of blood-brain barrier damage and so on. Yet, the conventional MRI approach may not be accurate enough when differentiating SBM from glioma, especially high-grade glioma (HGG), which presents as well-defined spacing occupying lesions with an enhancing rim in T1W + C accompanied by hyperintensity peritumoral edema in T2W [5]. And the morphological analysis suggested that the volume of tumor parenchyma, midline shift, and rim pattern in different conventional weighted MR sequences are either not optimal approaches for differentiating glioma from SBM [6, 7].

Over the years, advanced MRI technologies such as diffusion-weighted imaging (DWI), intravoxel incoherent motion (IVIM), and amide proton transfer-weighted (APTW) imaging have been developed and validated, and studies have shown that features extracted from those images can differentiate glioma from SBM [8–10]. DWI applies the diffusion gradient to characterize the mobility of water molecules in tissue when there is an inhibitory effect on cell membranes [11]. Intravoxel incoherent motion (IVIM) is a multi-b value DWI imaging that simultaneously measures the perfusion-associated microcirculation of blood capillaries at low-b values (b < 200 s/mm^2^) and the molecular water diffusion at high-b values. [12]. APTW imaging, as a relatively new noninvasive and endogenous contrast molecular imaging, utilizes the effect of chemical exchange saturation transfer between amide protons and water protons to measure the concentration of mobile proteins/peptides and tissue pH [13]. Various studies have shown that DWI, IVIM, APTW, or the combination of quantitative parameters have a higher diagnostic value than conventional MRI when assessing malignant tissue; they can quantify tumor regions and perform differentiation of different tumor types or even different tumor types or subtypes [14–17]. The histogram analysis based on MRI imaging, which could extract the characteristics based on quantitative tumor data distribution, was also investigated, and showed potential in glioma and SBM differentiation [18].

However, most current studies focused on the analysis of peritumoral or edema region for glioma and SBM [15, 17, 19] with histogram analysis dedicated to the structural weighted MRI image [20, 21] or quantile of functional image [22, 23]. Yet, it remains unclear whether the histogram features of the enhanced tumor area in advanced MRI can be helpful when assessing this type of tumor. Therefore, in this study, we used DWI, IVIM, and APTW imaging to investigate the differentiation of glioma and SBM with the parametric quantification and the histogram features.

## Methods

### Participants

**Fig. 1:**
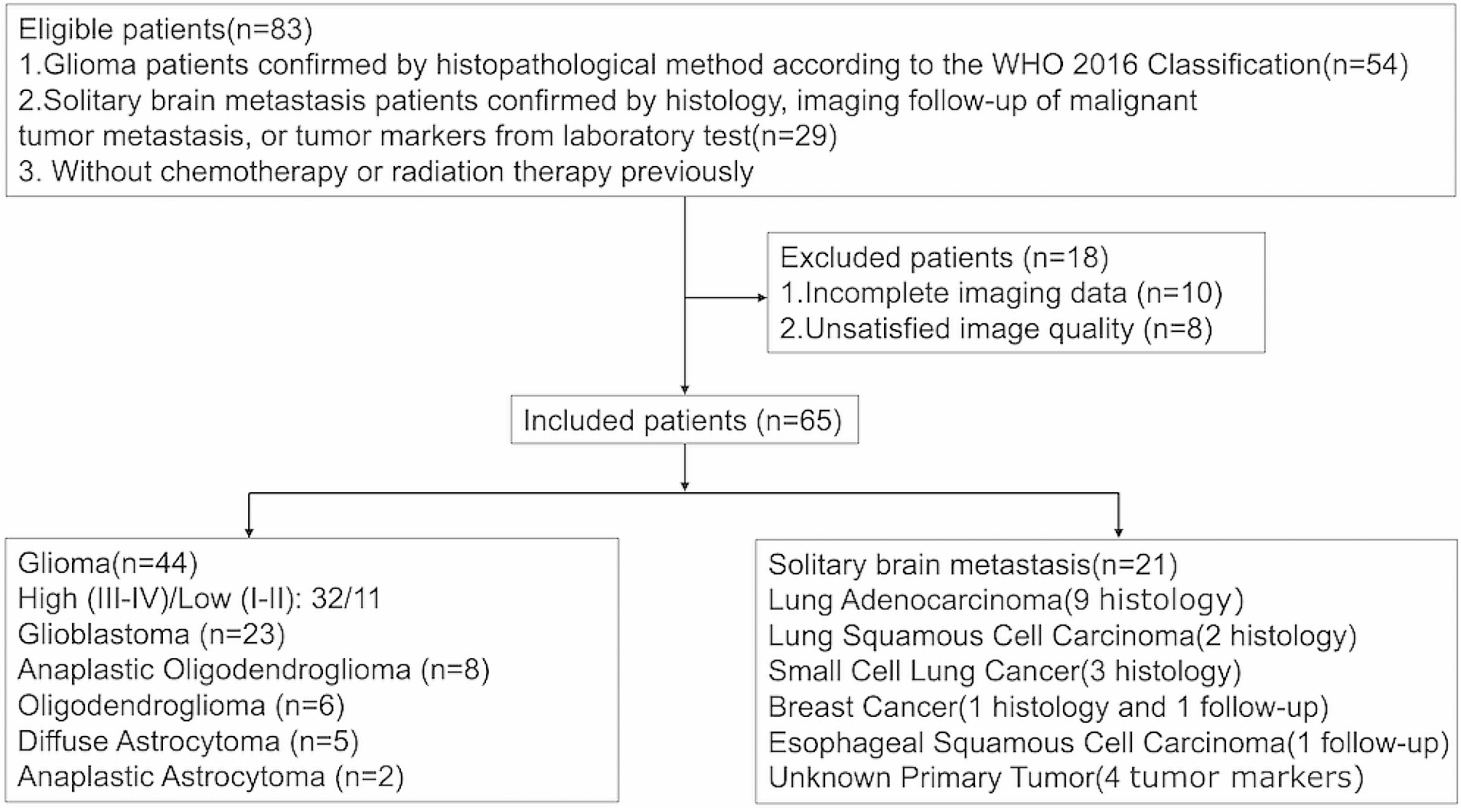
Study Flowchart

The protocol was reviewed and approved by the Ethics Committee of Shanxi Provincial People’s Hospital /Fifth Hospital of Shanxi Medical University (2022 Research Review No. 153). The informed consent was waived. All methods were carried out in accordance with the Declaration of Helsinki. A total of 83 patients with brain lesions were enrolled from December 2020 to May 2022. Inclusion criteria were: (1) patients with a definite diagnosis; (2) patients eligible for MRI, and have undergone MRI imaging preoperatively. Subjects who underwent chemotherapy or radiation therapy were excluded. All gliomas were diagnosed by surgical pathology (*n* = 54) according to the WHO 2016 Classification [24]; the diagnosis of SBM was obtained from histology (*n* = 29), imaging follow-up of malignant tumor metastasis (*n* = 2), or tumor markers from laboratory tests (*n* = 4) [25]. In addition, 18 patients were excluded prior to the analysis due to the following reasons: incomplete imaging data (*n* = 10) and/or unsatisfied image quality (i.e., significant cystic, hemorrhagic, or massive tumor necrosis; *n* = 8). The remaining 65 patients were included in the study, and the study flowchart is shown in Fig. 1.

### Data acquisition

MR sequences including T1W, T2W, T2 fluid-attenuated inversion recovery (FLAIR), IVIM, DWI, APT weighted imaging (APTWI), and contrast-enhanced T1W were conducted in a 3.0T MR scanner (Discovery MR 750 W, GE Healthcare, Waukesha, WI, USA) with a 24-channel head neck coil. Axial IVIM used 12 b-values (0, 20, 40, 80, 110, 150, 200, 400, 800, 1200, 1500, and 2000s/mm^2^ with respective averaging times 1, 1, 1, 1, 1, 1, 2, 2, 2, 2, 2, 2, 4). The following parameters were applied: repetition time/echo time (TR/TE) = 5400ms/90ms, the field of view (FOV) = 220 × 220mm^2^, matrix size = 110 × 110, slice thickness = 4.0 mm, number of slices = 24, slice gap = 1.0 mm, acquisition time = 5:28 min. The parameters for axial DWI with b-values of 0 and 1000s/mm^2^ were: TR/TE = 6000ms/76ms, FOV = 240 × 240mm^2^, matrix size = 120 × 120, slice thickness = 4.0 mm, number of slices = 29, slice gap = 1.0 mm. acquisition time = 0:48 min. Diffusion gradients were applied in three orthogonal directions for both IVIM and DWI, and the scanning slices covered the area of the whole lesion. The APTWI was acquired using a 2D single-shot fast spin-echo-based sequence (TR/TE = 2950ms/27ms, FOV = 256 × 256mm^2^, matrix size = 120 × 120, slice thickness = 8 mm, number of slices = 1, acquisition time = 2:06 min) with phased cycle pulses for saturation and the water saturation shift reference for B_0_ correction [26]. The total duration for phase cycle pulses is 2000ms under B1 of 2$\mu$T. The Z-spectra includes 52 frequencies, 49 of which offset from 600 to -600 Hz at an interval of 25 Hz, and three unsaturated images at 5000 Hz for signal normalization. During APTWI acquisition, we used the slice with the largest tumor diameter in the axial FLAIR as a reference while avoiding the slice with predominate hemorrhage or cyst. The acquisition parameters of T1W, T2W, FLAIR, and contrast-enhanced T1W were listed in Table S1. The total duration of the imaging protocol is about 30 min.

### Imaging processing

The DWI apparent diffusion coefficient (ADC) was generated on the Advantage Windows 4.7 workstation (General Electric Medical Systems) pixel by pixel with the formula:

1 $$\text{S}_\text{b}/\text{S}_\text{0}=\text{exp}(-\text{b}\times\text{ADC})$$

where S_b_ and S_0_ were the signal intensities for b = 1000 s/mm^2^ and b = 0 s/mm^2^. An in-house built program of IVIM bi-exponential fitting in MATLAB 2018b defined by (Eq. 2) was used for the calculation of slow diffusion coefficient (Dslow), fast diffusion coefficient (Dfast), and perfusion fraction (frac) within the region of interest (ROI).

2 $$\text{S}_\text{b}/\text{S}_\text{0}=\text{frac}\times\text{exp}\,(-\text{b}\times\text{Dfast})+(1-\text{frac})\times\text{exp}\,(-\text{b}\times\text{Dslow})$$

where S_b_ and S_0_ were the signal intensities for b ranging from 0 to 1200 s/mm^2^ and b = 0 s/mm^2^. A linear fitting was first applied to the logarithm of the diffusion data with b ≥ 200s/mm^2^ to obtain the D parametric map, after which all the diffusion data (b = 0 ∼ 1200 s/mm^2^) were used to fit the bi-exponential model (Eq. 2) with a bound-constrained optimization mini-search method using the online MATLAB code [27]. The APTWI asymmetric magnetization transfer ratio at 3.5 ppm is represented as MTRasym@3.5 ppm and calculated by:

3 $$\text{MTRasyam@}3.5\,\text{ppm}=[\text{S}_\text{sat}(-3.5\,\text{ppm})-\text{S}_\text{sat}(+3.5\,\text{ppm})]/\text{S}_\text{0}$$

using the vendor provided post-processing program, where S_0_ is non-saturation intensity while S_sat_ is the signal intensity after saturation.

A neuro-radiologist with 20 years of MR imaging reading experience, Y.-X.H who was blinded to the definite diagnosis, drew the ROI of tumor parenchyma on images of IVIM b = 0 s/mm^2^, and maps of DWI ADC and APTWI MTRasym@3.5 ppm with contrast-enhanced T1W image as the reference where the cerebrospinal fluid-filled, calcification, hemorrhagic, necrotic, and cystic areas were avoided wherever possible in 3D-Slicer (https://www.slicer.org/, version 4.10). Three-dimensional ROI was generated for IVIM parametric maps (frac, Dfast, Dslow) and DWI ADC while a 2-dimensional ROI was obtained on the only one acquired slice for APTWI. The histogram features, including mean, 10th percentile, 90th percentile, entropy (a measure of the disorder of a distribution), kurtosis (a measure of the tailedness of a distribution), and skewness (a measure of the asymmetry of a distribution) for all parametric maps in tumor ROIs were extracted with an in-house built program in MATLAB 2018b according to the formula on website (https://pyradiomics.readthedocs.io/en/latest/features.html).

### Statistical analysis

Quantitative variables were expressed as the mean ± standard deviation and were compared with the Student’s t-test or the Wilcoxon test (Mann-Whitney *U* test) after the normality and homogeneity of variance were confirmed. The binary data for clinical information were compared using the Chi-Squared test. Subsequently, variables exhibiting significant differences but not strong correlations were evaluated using both univariate and multivariate logistic regression. Receiver Operating Characteristic (ROC) analysis was then employed to assess the performance of individual factors and multi-parameter combined models in differentiating glioma from SBM. The area under the ROC curve (AUC), sensitivity, specificity, positive predictive value (PPV), and negative predictive value (NPV) were calculated and compared. Nomogram and bootstrap resampling methods were used for the evaluation of the multivariate logistics regression. R package (version 4.0.0) was used for all the statistics. A P value < 0.05 indicated a statistically significant difference.

## Results

### Clinical characteristics

A number of 65 patients (44 glioma cases with age 51.27 ± 13.09 and 21 SBM cases with age 59.05 ± 12.33) were finally included in this study. Demographics and clinical manifestation are summarized in Table 1. The age in the SBM group was significantly greater than that of the glioma group, while no significant difference was found for gender and clinical manifestations (secondary epilepsy, intracranial hypertension, and dysfunction) between groups.

**Table 1:** Clinical characteristics

	Glioma group (*n* = 44)	SBM group (*n* = 21)	*P*
**Demographics**			
Male (n, %)	25 (56.8%)	10 (47.6%)	0.615
Age at diagnosis (year)	51.27 ± 13.09	59.05 ± 12.33	0.019*
**Clinical manifestations**			
Secondary epilepsy (n, %)	18 (40.9%)	4 (19.5%)	0.105
Intra-cranial hypertension (n, %)	22 (50.0%)	11 (52.4%)	1
Dysfunction (n, %)	18 (40.9%)	14 (66.7%)	0.065

* *P* < 0.05. Data are statistically significant

### Comparison of histogram features between glioma and SBM

**Fig. 2:**
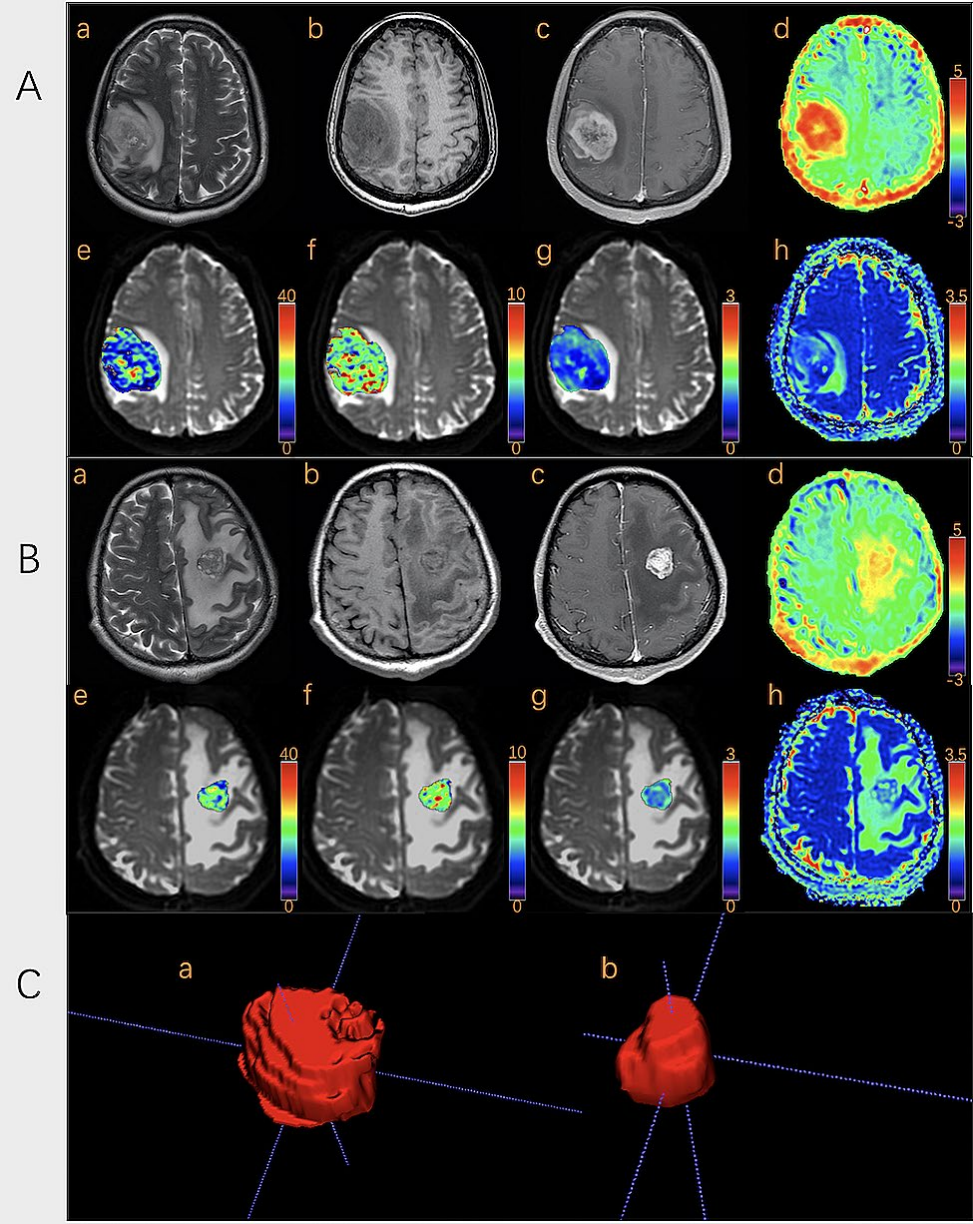
Representative images of patients with glioma or metastases. (Row **A** & Row **B**): **a**: T2W, **b**: T1W, **c**: contrast-enhanced T1W, **d**: MTRasym@3.5ppm, **e**: Dfast(×10^− 3^mm^2^/s), **f**: Dslow(×10^− 3^mm^2^/s), **g**: frac (%), **h**: ADC(×10^− 3^mm^2^/s). Row **C**: 3D reconstruction of tumor ROI of glioma patient (**a**) and SBM patient (**b**). Row **A**: A 63-year-old woman pathologically confirmed with glioblastoma. MRI showed an irregular lesion with unclear margin in the right frontal lobe, presenting as a diffuse enhanced lesion with hyper-intensity on T2W and hypo-intensity on T1W accompanied by peritumoral edema. Histogram features of the enhanced tumor parenchyma are as follows: ADC_kurtosis_:4.84. (MTRasym@3.5ppm)_10_: 1.5, frac_10_:7.38, frac_90_:18.79, frac_mean_:12.85, frac_entropy_:3.26, frac_kurtosis_:4.92, frac_skewness_:0.90. Row **B**: A 34-year-old woman was pathologically confirmed with adenocarcinoma; a primary lesion was found in her left lung. MRI showed a regular lesion with a clear margin in the left frontal lobe, presenting as a diffuse-enhanced lesion with iso-intensity on T2W and T1W accompanied by peritumoral edema. Histogram features of the enhanced tumor parenchyma are as follows: ADC_kurtosis_:2.74. (MTRasym@3.5ppm)_10_: 1.9, frac_10_:12.62, frac_90_:21.94, frac_mean_:17.21, frac_entropy_:3.95, frac_kurtosis_:3.22, frac_skewness_:0.13

**Fig. 3:**
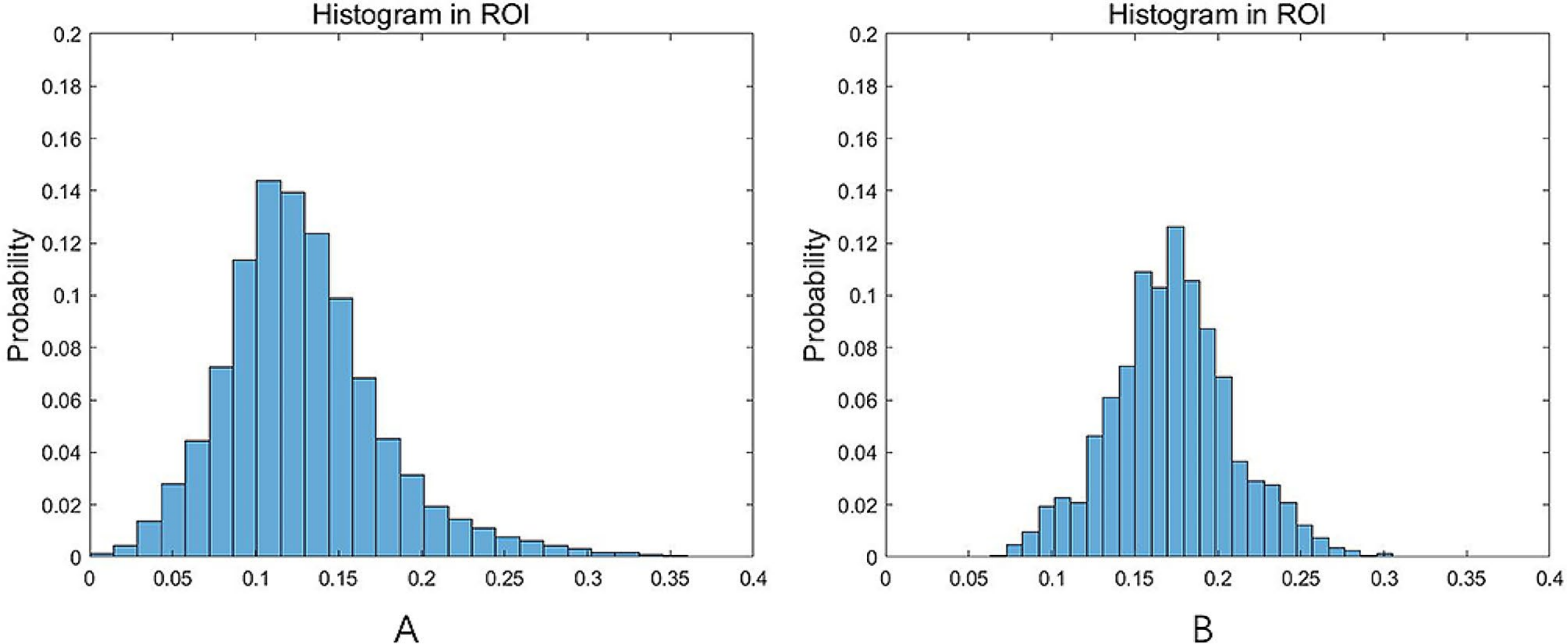
Histogram of frac in the enhanced tumor region. The histogram showed that the glioma patient (**A**) has higher frac_kurtosis_, and frac_skewness_ but lower frac_entropy,_ than the SBM patient (**B**) in the enhanced tumor parenchyma region

Significant differences for ADC_kurtosis_ (6.43 ± 5.63 vs. 4.54 ± 3.23, *P* = 0.022), (MTRasym@3.5ppm)_10_ (0.53 ± 0.97 vs. 1.00 ± 0.75, *P* = 0.045), frac_10_ (6.81 ± 2.07 vs. 9.34 ± 2.75, *P*<0.001), frac_90_ (23.90 ± 6.22 vs. 29.73 ± 6.47, *P* = 0.001), frac_mean_ (14.67 ± 3.64 vs. 18.76 ± 4.10, *P*<0.001), frac_entropy_ (3.72 ± 0.35 vs. 4.06 ± 0.24, *P*<0.001), frac_kurtosis_ (5.37 ± 2.41 vs. 3.21 ± 0.83, *P*<0.001), and frac_skewness_ (1.05 ± 0.55 vs. 0.50 ± 0.39, *P*<0.001) were observed between glioma and SBM, while other quantitative values showed no significant differences **(**Table 2**)**. Representative images of one patient with glioma and another with SBM are shown in Fig. 2. Histograms of frac in the same patient are shown in Fig. 3.

**Table 2:** Comparison of parametric values in the region of interest between glioma and SBM

	10th percentile	90th percentile	mean	entropy	kurtosis	skewness
ADC (×10^− 3^mm^2^/s)						
Glioma	0.85 ± 0.16	1.51 ± 0.34	1.16 ± 0.22	5.23 ± 0.52	6.43 ± 5.63	0.98 ± 0.93
SBM	0.80 ± 0.19	1.58 ± 0.28	1.15 ± 0.21	5.37 ± 0.50	4.54 ± 3.23	0.87 ± 0.74
p	0.253	0.454	0.784	0.164	0.022*	0.563
MTRasym@ 3.5 ppm						
Glioma	0.53 ± 0.97	2.23 ± 1.31	1.40 ± 0.98	0.58 ± 0.51	3.08 ± 1.13	-0.10 ± 0.42
SBM	1.00 ± 0.75	2.39 ± 0.85	1.66 ± 0.70	0.58 ± 0.47	3.39 ± 1.14	0.20 ± 0.62
p	0.045*	0.622	0.465	0.725	0.238	0.057
Dfast (×10^− 3^mm^2^/s)						
Glioma	2.80 ± 0.44	8.04 ± 1.76	5.11 ± 0.81	3.78 ± 0.38	5.97 ± 3.57	1.31 ± 0.42
SBM	3.03 ± 0.48	7.69 ± 1.81	5.19 ± 0.79	3.75 ± 0.39	5.91 ± 2.49	1.13 ± 0.61
p	0.056	0.369	0.884	0.717	0.972	0.229
frac (%)						
Glioma	6.81 ± 2.07	23.90 ± 6.22	14.67 ± 3.64	3.72 ± 0.35	5.37 ± 2.41	1.05 ± 0.55
SBM	9.34 ± 2.75	29.73 ± 6.47	18.76 ± 4.10	4.06 ± 0.24	3.21 ± 0.83	0.50 ± 0.39
p	< 0.001*	0.001*	< 0.001*	< 0.001*	< 0.001*	< 0.001*
Dslow (×10^− 3^mm^2^/s)						
Glioma	0.77 ± 0.15	1.33 ± 0.26	1.04 ± 0.20	3.92 ± 0.31	4.12 ± 2.88	0.61 ± 0.62
SBM	0.73 ± 0.13	1.32 ± 0.25	1.00 ± 0.17	4.05 ± 0.30	3.52 ± 1.77	0.64 ± 0.62
p	0.248	0.959	0.465	0.063	0.182	0.702

* *P* < 0.05. Data are statistically significant

### The diagnostic performance of histogram features

The regression analysis showed that frac_10_ (OR = 1.573, 95%CI 1.179–2.099, *P* = 0.002), frac_90_ (OR = 1.147, 95%CI 1.048–1.256, *P* = 0.003), frac_mean_ (OR = 1.304, 95% CI 1.11–1.533, *P* = 0.001), frac_entropy_ (OR = 29.620, 95% CI 3.753–233.8, *P* = 0.001), frac_kurtosis_ (OR = 0.431, 95% CI 0.256–0.723, *P* = 0.002), and frac_skewness_ (OR = 0.1, 95% CI 0.026–0.389, *P* < 0.001) were the associated factors for glioma and SBM differentiation, but only the frac_kurtosis_ was an independent factor (Table 3, Table S2).

**Table 3:** Univariate and multivariate regression analysis

	Univariate			Multivariate-1^†^			Multivariate-2^‡^		
	OR	(95% CI)	*P*	OR	(95% CI)	*P*	OR	(95% CI)	*P*
ADC_kurtosis_	0.887	(0.741–1.062)	0.193						
(MTRasym @3.5ppm) _10_	1.892	(0.954–3.754)	0.068	2.084	(0.931–4.664)	0.074			
frac_10_	1.573	(1.179–2.099)	0.002*	1.29	(0.97–1.717)	0.081	1.279	(0.961–1.702)	0.092
frac_90_	1.147	(1.048–1.256)	0.003*						
frac_mean_	1.304	(1.11–1.533)	0.001*						
frac_entropy_	29.620	(3.753–233.8)	0.001*						
frac_kurtosis_	0.431	(0.256–0.723)	0.002*	0.492	(0.272–0.891)	0.019*	0.507	(0.289–0.889)	0.018*
frac_skewness_	0.1	(0.026–0.389)	< 0.001*						

^†^Multivariate-1(Multivariate regression model 1): (MTRasym@3.5ppm)_10_+ frac_10_ + frac_kurtosis_

^‡^Multivariate-2(Multivariate regression model 2): frac_10_ + frac_kurtosis_

* *P* < 0.05. Data are statistically significant

**Fig. 4:**
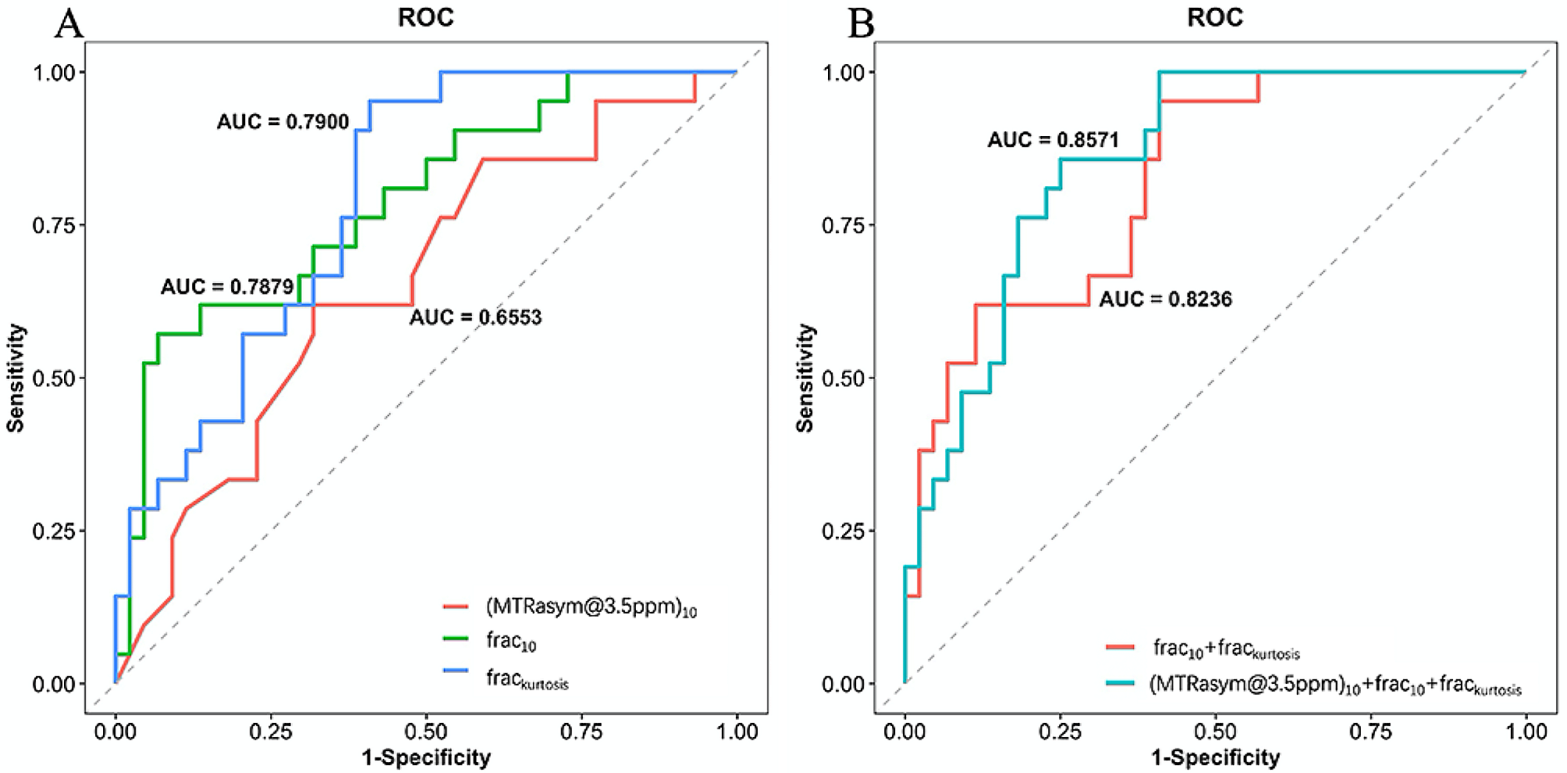
The ROC curves for univariate (**A**) and multivariate regression (**B**)

Better sensitivity of frac_kurtosis_ (0.952) and better specificity of frac_10_ (0.932) were found by ROC analysis compared with other associated factors (Table 4; Fig. 4A). And frac_kurtosis_ demonstrated the best AUC (0.790). Improved diagnostic AUC (AUC = 0.857, accuracy = 0.785, sensitivity = 0.857, specificity = 0.750, PPV = 0.621, NPV = 0.917) can be obtained when combining the (MTRasym@3.5ppm)_10_, frac_10_ and frac_kurtosis_. The model integrating the frac_10_ and frac_kurtosis_ was also evaluated, and the AUC was 0.824 (accuracy = 0.708, sensitivity = 0.952, specificity = 0.591, PPV = 0.526, NPV = 0.963) (Table 4; Fig. 4B).

**Table 4:** The univariate and multivariate performance in the differentiation of glioma and metastases^†^

	AUC (95% CI)	Sensitivity (95% CI)	Specificity (95% CI)	Accuracy (TN + TP)/(*N* + *P*)	PPV (TP/*P*)	NPV (TN/*N*)	Threshold
**Univariate**							
(MTRasym @3.5ppm) _10_	0.655 (0.513–0.798)	0.714 (0.524–0.905)	0.682 (0.523–0.818)	0.662 (43/65)	0.481 (13/27)	0.789 (30/38)	0.905
frac_10_	0.788 (0.667–0.909)	0.571 (0.381–0.762)	0.932 (0.841-1)	0.815 (53/65)	0.8 (12/15)	0.82 (41/50)	9.253
frac_90_	0.76 (0.635–0.884)	0.952 (0.857-1)	0.5 (0.363–0.636)	0.646 (42/65)	0.476 (20/42)	0.957 (22/23)	21.913
frac_mean_	0.784 (0.664–0.903)	0.762 (0.571–0.952)	0.727 (0.591–0.841)	0.738 (48/65)	0.571 (16/28)	0.865 (32/40)	16.249
frac_entropy_	0.767 (0.65–0.885)	0.952 (0.857-1)	0.613 (0.477–0.75)	0.723 (47/65)	0.541 (20/37)	0.964 (27/28)	3.842
frac_kurtosis_	0.790 (0.682–0.898)	0.952 (0.857-1)	0.591 (0.454–0.727)	0.708 (46/65)	0.526 (20/38)	0.963 (26/27)	4.659
frac_skewness_	0.784 (0.671–0.896)	0.714 (0.524–0.905)	0.750 (0.614–0.864)	0.738 (48/65)	0.577 (15/26)	0.846 (33/39)	0.728
**Multivariate** ^†^							
Multivariate-1^‡^	0.857 (0.769–0.946)	0.857 (0.714-1)	0.750 (0.614–0.864)	0.785 (51/65)	0.621 (18/29)	0.917 (33/36)	0.304
Multivariate-2^§^	0.824 (0.720–0.927)	0.952 (0.858-1)	0.591 (0.431–0.727)	0.708 (46/65)	0.526 (20/38)	0.963 (26/27)	0.192

^†^DeLong’s test showed no statistically significant differences between AUCs from two multivariate regression models. (Z = -1.14, *P* = 0.25) and McNamar’s test revealed statistically significant differences in specificities (*P* = 0.016) but not in sensitivities (*P* = 0.5) from two multivariate regression models

^‡^Multivariate-1(Multivariate regression model 1): (MTRasym@3.5ppm)_10_+ frac_10_ + frac_kurtosis_

^§^Multivariate-2(Multivariate regression model 2): frac_10_ + frac_kurtosis_

AUC: area under the curve; PPV: Positive predictive value; NPV: negative predictive value; P: positives; N: negatives; TP: true positives; TN: true negatives

No significant difference was found for AUC between these two combined models by the Delong test (Z = -1.14, *P* = 0.25), but the McNemar test revealed statistically significantly improved specificity (*P* = 0.016) for model including (MTRasym@3.5ppm)_10_. The sensitivity from two multivariate regression models showed no significant difference (*P* = 0.5). The Nomogram and calibration analysis showed a good correlation between apparent and bias-corrected multivariate logistic regression in both models (Figure S1).

## Discussion

Histogram analysis of parameters acquired from DWI, IVIM, and APTWI were applied to differentiate glioma and SBM. Significantly lower (MTRasym@3,5ppm)_10_, frac_10_, frac_90_, frac_mean_, frac_entropy_ and significantly higher ADC_kurtosis_ frac_kurtosis_ and frac_skewness_ were found in the parenchyma area of glioma compared to that of SBM. The combined model with (MTRasym@3.5ppm)_10_, frac_10_ and frac_kurtosis_ showed the optimal AUC performance and superior specificity.

As a noninvasive method for tumor differentiation, MRI is effective but dilemmatic sometimes when discriminating glioma, especially high-grade glioma, and SBM [28, 29]. Glioma grows infiltratingly in the brain, whereas SBM grows in an expansive pattern, the differences in growth patterns may imply differences in microstructure and cell properties. Previous MR and pathology studies have shown that ADC was well correlated with cell intensity [30, 31]. A more positive kurtosis indicates more data around the mean, while the lower kurtosis values in the SBM group indicate more inhomogeneous ADC values around the mean. The same level of ADC percentile and mean value between glioma and SBM found in the current study suggested that it may be challenging to differentiate them from the cell density. A higher ADC_kurtosis_ in glioma also indicates more outliers at the ends of the ADC distribution, meaning that gliomas have more voxels with extremely large or small ADC values than SBM. Correspondingly, water diffusion in glioma patients is more affected by the microstructure. Gao et al. [23] showed that maximum fractional anisotropy was significantly higher in glioblastoma than in SBM in the enhanced tumor area. In addition, Mao et al. found significantly lower isotropic volume fraction and orientation dispersion index from neurite orientation dispersion and density imaging of the contrast-enhancing tumors in glioma tissue in comparison with SBM [32]. These results suggest that glioma might have a more complex microstructure. Consequently, more water molecule activity is affected, which leads to higher ADC kurtosis in glioma.

Neovascularization is a critical mechanism in tumor growth and metastasis that transports nutrients and removes metabolic waste from tumor cells. And varied tumor cell distributions may result in varied angiogenesis. IVIM perfusion fraction is the ratio of micro-vascular diffusion to total micro-vascular and molecular water diffusion, both of which were associated with micro-structure [33]. Smaller frac_kurtosis_ value and greater frac_entropy_ in SBM were consistent with the representative histogram in Fig. 3. From the ADC kurtosis analysis, it could be inferred that the distribution of frac in glioma was more concentrated and less outliers at the ends of the frac distribution, meaning that gliomas have less voxels with extremely large or small frac values than SBM, correspondingly, smaller frac_entropy_ in glioma. The significant difference in frac_entropy_ but not in ADC_entropy_ means the heterogeneity of relative change in microvascular perfusion and water diffusion may be a more sensitive feature to differentiate glioma from SBM. Larger frac_skewness_ in glioma means the ratio of voxels with a high degree of frac was larger than that of SBM, meaning that glioma has more micro-vascular diffusion free area. But lower frac_mean_ value in glioma in our current study may reflect a micro-vessel perfusion restriction phenomenon in the whole tumor area. Study by Heynold et al. showed that glioblastomas have higher neovascularization activity and metabolic rate of oxygen in enhancing area compared with that of brain metastasis [34], while microscopic intravascular thrombosis driven by the neoplastic overexpression of pro-coagulants could attenuate glioma blood supply [35], causing a perfusion-limited hypoxia, consequently. Furthermore, Shim et al. [14] investigated the differentiation of glioblastoma and brain metastasis with IVIM and found no significant difference in any of quantitative parameters between the two kinds of lesions. One reason for the difference may result from the adopted b values [36, 37], which ranged from 0 s/mm^2^ to 900 s/mm^2^ in [14] vs. 0 s/mm^2^ to 1200 s/mm^2^ in the present study. On the other side, five small-size ROIs with the hot-spot method in enhanced areas were used in [14] while we delineated all the enhanced areas.

Built upon noticeable disparities in diffusion and micro-perfusion traits between gliomas and SBM, we can deduce the existence of a hypoxic tumor microenvironment specific to gliomas. Recent scholarly inquiries underscore the profound influence of hypoxia on cancer cells, intricately impacting behavior, treatment responses, and prognostic outcomes, and the intricate interplay encompasses a diverse spectrum of signaling pathways and gives rise to discernibly distinct protein expression patterns [38–40]. MTRasym@3.5ppm from APTWI was used to quantify the percent signal generated from mobile proteins and peptides [41]. A two-dimensional APTWI study showed that the mean, 10th, 25th, 50th, 75th, and 90th percentile of MTRasym@3.5ppm in enhancing areas of glioblastomas were significantly higher than those of SBM, and the 10th percentile was used to obtain the optimal AUC (0.85) for glioma and SBM differentiation [22]. Another study claimed that the maximum, minimum, and mean of MTRasym@3.5ppm in the tumor core did not significantly differ between the SBM and GBM [19]. In our study, the 10th percentile was significantly lower in glioma than in SBM, but the differentiation performance was relatively low (AUC = 0.655, sensitivity = 0.714, and specificity = 0.682). And other histogram features showed no differences. Studies have revealed that the high-grade glioma has higher MTRasym@3.5 ppm [42–44] and the enrollment of low-grade glioma in the present study may have an impact on the result.

Considering the distinct growth patterns of glioma and brain metastasis—glioma exhibits infiltrative growth beyond the boundaries of the enhancing tumor core, while brain metastasis grows expansively—the peritumoral edema may manifest differently in diffusion and perfusion characteristics. Literature review revealed higher perfusion values in the peritumoral edema area of glioma compared to brain metastasis [45–47], along with a decreasing gradient of relative cerebral blood volume values from the region adjacent to the enhancing solid lesion to the normal white matter in glioma; however, this gradient is less pronounced in brain metastasis [48–50]. Given the limited availability of pertinent literature in diffusion imaging, we intend to undertake a comparative investigation employing the IVIM method aiming to elucidate intricate details of diffusion and perfusion characters between the two tumor types in our further study.

In multivariate regression analysis, models with frac_10_ and frac_kurtosis_ provided optimal differentiation performance, and (MTRasym@3.5ppm)_10_ did not affect the abilities of combined models. Though the AUC (0.857, 0.824) is less than the quantification with ADC of high b value [17] and APTWI [19] in edema and peritumoral region, it was comparable to the result in [22] with MTRasym@3.5ppm percentiles and better than the result in [32] with multiple advanced diffusion models in enhanced tumor core area. Multivariant 1 model has marginally higher AUC (*P* = 0.25) than Multivariant 2 model at cost of lower sensitivity (*P* = 0.5) but much better specificity (*P* = 0.016). When making surgical decisions, the assessment of a patient’s suitability for the procedure and the potential perioperative complications plays a critical role. Younger patients, typically with fewer underlying medical conditions, tend to benefit more from surgical interventions. For gliomas, proactive surgical approaches can significantly extend overall survival, emphasizing the importance of diagnostic sensitivity. Conversely, older patients, often with reduced surgical tolerance, require a higher degree of diagnostic specificity to accurately rule out a metastatic tumor diagnosis.

In our present study, IVIM bi-exponential fitting model with b-values between 0 and 1200 s/mm^2^ was used for analysis, for a poor fit in our initial analysis at b = 1500 and 2000 s/mm^2^. And various b-value protocols have been used for the IVIM analysis, but there is no standard setting [37, 51]. Further, the IVIM signal may be affected by other factors. The study by Hare et al. [52]. indicated that when cerebrospinal fluid was nullified, Dfast values showed a significant decrease, and a mono-exponential model was sufficient to describe the diffusion signal in the brain. This observation suggested that IVIM is more sensitive to cerebrospinal fluid than to brain microvasculature. In contrast, Rydhog et al. [53]. reported that both free water and vascular fractions are measurable in brain tissue. In conclusion, there are varying degrees of controversy in IVIM research, spanning from sequence parameter settings to signal analysis and data interpretation. Further investigation is required in these domains.

There are some limitations in the present study. First, the number of enrolled subjects is relatively small, and only part of the subtypes was analyzed, i.e., the brain metastasis resulted mainly from the lung lesions, and the glioma in all grades was included. And the imbalance between the two classes may cause bias in analysis. Second, we delineated tumor ROIs at multiple layers on the parametric maps of ADC and IVIM but only the slice with the largest tumor parenchyma on 2-dimensional APTWI. And all the ROIs were drawn by one reader. Both may have an impact on the analysis. And In addition, detailed pathological information such as immunohistochemical staining, cell distribution density, and micro-vessel density was not used as the reference, which may help confirm these findings and should be considered in the future.

## Conclusions

The frac_10_ and frac_kurtosis_ from IVIM MRI in enhanced tumor region demonstrated the difference between glioma and solitary brain metastasis and (MTRasym@3.5ppm)_10_ helps improving the differentiating specificity. And their combing model could be used as a useful imaging biomarker for differentiation.

### Electronic supplementary material

Below is the link to the electronic supplementary material.

Supplementary Material 1

## Data Availability

The datasets used and analyzed during the current study are available from the corresponding author on reasonable request….

## Abbreviations

ADC: Apparent diffusion coefficient
ADC_kurtosis_: Kurtosis of ADC
APTWI: Amide proton transfer-weighted imaging
AUC: Area under the curve
DWI: Diffusion-weighted imaging
Dslow: Slow diffusion coefficient
Dfast: Fast diffusion coefficient
frac: Perfusion fraction
frac_10_, frac_90_, frac_mean_, frac_kurtosis_, frac_entropy_, frac_skewness_: 10th percentile,90th percentile, mean, kurtosis, and entropy of perfusion fraction
HGG: High-grade glioma
IVIM: Intravoxel incoherent motion
MRI: Magnetic resonance imaging
MTRasym@3.5 ppm: Asymmetric magnetization transfer ratio at 3.5 ppm
(MTRasym@3.5 ppm) _10_: 10th percentile of MTRasym@3.5 ppm
OR: Odds ratio
ROC: Receiver operation characteristic
ROI: Region of interest
SBM: Solitary brain metastasis
T1W: T1 weighted
T1W + C: Contrast-enhanced T1W
T2W: T2 weighted
