# Supplementary material for: Differentiation of glioma and solitary brain metastasis: a multi-parameter magnetic resonance imaging study using histogram analysis
Source: BMC Cancer. 2024 Jul 5;24:805. doi: 10.1186/s12885-024-12571-5 (PMC11225204; doi:10.1186/s12885-024-12571-5)
Supplement: Supplementary file 1 — Supplementary Material 1 [file 12885_2024_12571_MOESM1_ESM.docx]

**Supplementary files**

**Table S1.** The common parameters of other MR sequences used in the study

|  | T2W (axial) | FLAIR (axial) | T1W (axial) | T1W + C* |
| --- | --- | --- | --- | --- |
| TR/TE (ms/ms) | 7900/125 | 9000/100 | 2500/24 | 2500/24 |
| FOV (mm^2^) | 230×230 | 230×230 | 230×230 | 230×230 |
| matrix size | 380×380 | 260×200 | 320×224 | 320×224 |
| slice thickness (mm) | 4.5 | 4.5 | 4.0 | 4.0 |
| number of slices | 30 | 30 | 30 | 30 |
| gap (mm) | 5 | 5 | 5 | 5 |
| flip angle (°) | 140 | 160 | 110 | 110 |
| acquisition time (min) | 2:00 | 2:52 | 1:19 | 1:19 |

T2W: T2 Weighted, FLAIR: fluid-attenuated inversion recovery, T1W: T1 Weighted, T1W + C: Contrast-enhanced T1W, TR/TE: repetition time/echo time, FOV: the field of view, min: minute

*For T1W + C, gadodiamide (OMNISCAN^®^, GE Healthcare, USA) was administered was power-injected at doses standardized according to the patient’s body weight (0.2 ml/kg) at 2 ml/s, and the scan was started at 3–5 min after injection. Images on axial, sagittal and coronal planes were obtained in succession.

**Table S2.** Correlation coefficients between quantitative values in the regions of interest

|  | ADC_kurtosis_ | (MTRasym  @3.5ppm) _10_ | frac_10_ | frac_90_ | frac_mean_ | frac_entropy_ | frac_kurtosis_ | frac_skewness_ |
| --- | --- | --- | --- | --- | --- | --- | --- | --- |
| ADC_kurtosis_ | 1 | -0.039 | -0.315 | -0.225 | -0.303 | -0.303 | 0.3 | 0.245 |
| (MTRasym  @3.5ppm) _10_ |  | 1 | 0.124 | 0.026 | 0.059 | 0.002 | -0.105 | -0.179 |
| frac_10_ |  |  | 1 | 0.672 | 0.841 | 0.521 | -0.657 | -0.766 |
| frac_90_ |  |  |  | 1 | 0.949 | 0.9 | -0.82 | -0.619 |
| frac_mean_ |  |  |  |  | 1 | 0.821 | -0.829 | -0.741 |
| frac_entropy_ |  |  |  |  |  | 1 | -0.881 | -0.668 |
| frac_kurtosis_ |  |  |  |  |  |  | 1 | 0.867 |
| frac_skewness_ |  |  |  |  |  |  |  | 1 |

**
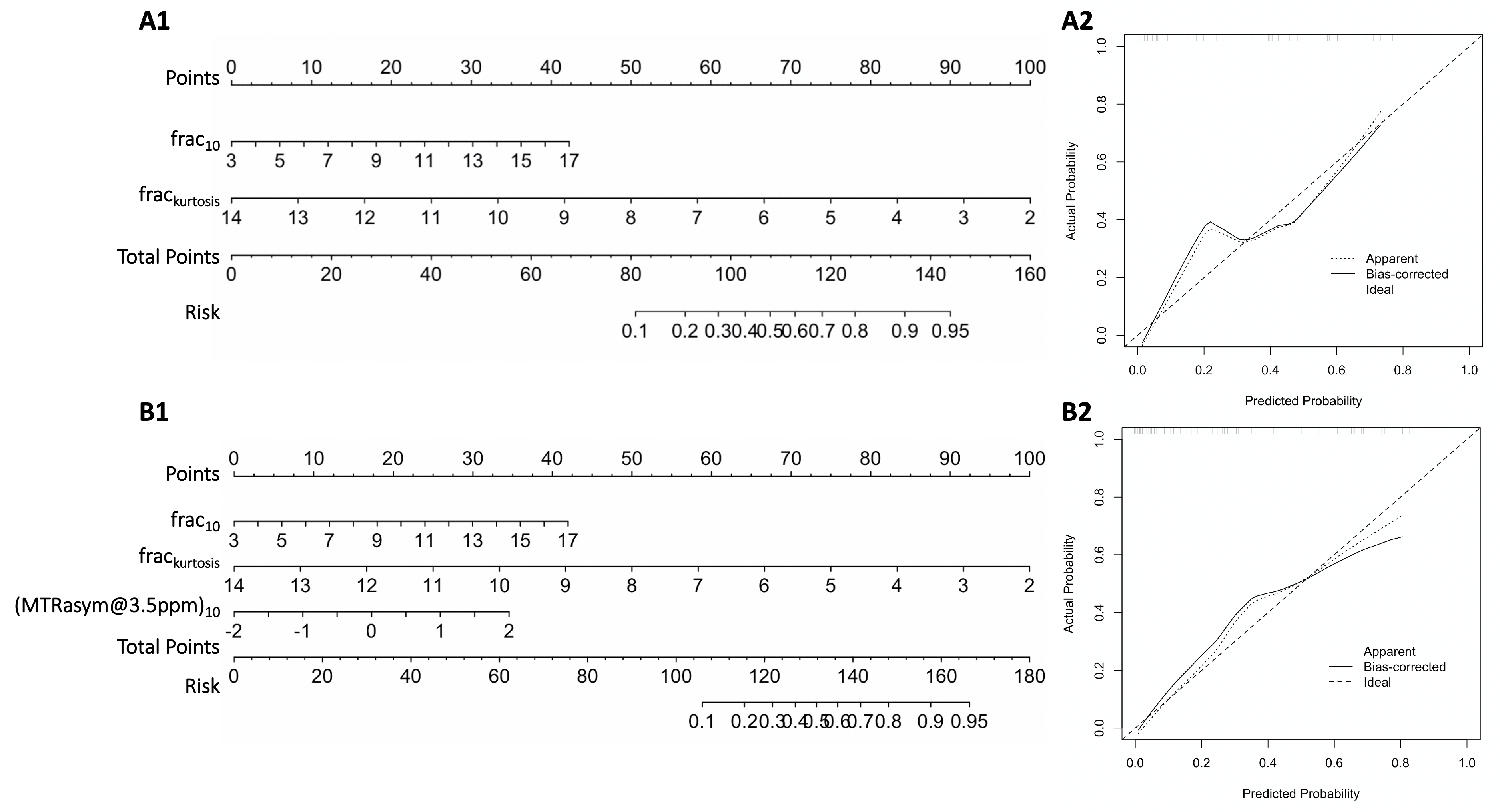
**

**Figure S1. Nomogram and calibration analysis of multivariate analysis.** *Nomogram of Multivariate-1 (Multivariate regression model 1): frac_10_ + frac_kurtosis_ and Multivariate-2 (Multivariate regression model 2): (MTRasym@3.5ppm)_10_ + frac_10_ + frac_kurtosis_(B1). Calibration analysis with bootstrap resampling method of Multivariate-1 (A2) and Multivariate-2 (B2). For Multivariate-1, Pearson’s r = 0.940 (95% CI: 0.896-0.966, P< 0.001), Intraclass correlation coefficient (ICC) =0.925 (95% CI: 0.872-0.957, P<0.001). For Multivariate-2, Pearson’s r = 0.973 (95% CI: 0.952-0.984, P<0.001), Intraclass correlation coefficient (ICC) =0.956 (95% CI: 0.924-0.975, P<0.001).*
